# Role of constituents for the chirality isolation of single-walled carbon nanotubes by the reversible phase transition of a thermoresponsive polymer†

**DOI:** 10.1039/d0ra04357e

**Published:** 2020-06-29

**Authors:** Eriko Shimura, Tomomi Tanaka, Yuki Kuwahara, Takeshi Saito, Toshiki Sugai, Shota Kuwahara

**Affiliations:** Department of Chemistry, Faculty of Science, Toho University 2-2-1 Miyama Funabashi Chiba 274-8510 Japan syouta.kuwahara@sci.toho-u.ac.jp; Nanomaterials Research Institute, National Institute of Advanced Industrial Science and Technology Tsukuba 305-8565 Japan

## Abstract

The simple sorting procedure and continuous use of poly(*N*-isopropylacrylamide) (PNIPAM), a well-known thermoresponsive polymer, have a high potential for the mass production of single-walled carbon nanotubes (SWCNTs) with a specific electronic structure. However, knowledge of efficient single-chirality sorting methods with mixed surfactant systems is not applicable. In this work, we explored experimental conditions by controlling the interaction among PNIPAM, sodium cholate (SC) and SWCNTs. An optimization of the PNIPAM and SC concentrations as well as the addition of sodium borate achieved the selective release of (6,4) nanotubes into the liquid phase after the PNIPAM phase transition. The sorting mechanism with PNIPAM was explained by the difference in the micelle configuration on the SWCNTs and the hydrophobic collapse of PNIPAM in the presence of a sodium salt. The one-step sorting procedure for obtaining SWCNTs with a single chirality *via* PNIPAM will help promote their widespread application.

## Abstract 2

Optimized experimental conditions in the presence of sodium borate achieved the selective release of (6,4) nanotubes into the liquid phase.

## Graphical abstract

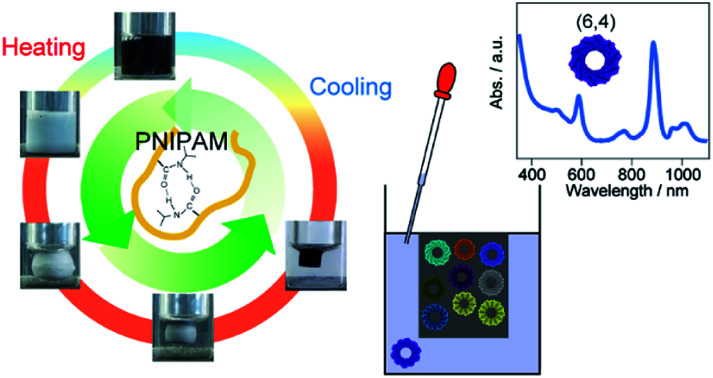

## Introduction

Single-walled carbon nanotubes (SWCNTs) have unique electronic and optical properties closely related to their chirality, which is defined by a pair of integers (*n*,*m*) that indicates the direction in which the graphite sheet is rolled to form the nanotube cylinder. An ultimate SWCNT nanotechnology will be achieved by using structurally pure SWCNTs with a single chirality, which paves the way for pioneering the frontier of nanotechnology application.^1–4^ Mass production of SWCNTs with a single chirality is still a challenging task for researchers in the field of nanomaterials. One promising candidate for obtaining SWCNTs with a single chirality is the bulk sorting of as-synthesized SWCNTs, including structural heterogeneity.

Since M. J. O'Connell and coworkers succeeded in the preparation of individual SWCNTs in aqueous micellar suspensions *via* ultrasonication with a surfactant, various sorting techniques for obtaining electronically homogenous SWCNTs have been achieved.^5^ Most dispersion-based sorting methods, such as density gradient ultracentrifugation (DGU),^6–8^ column chromatography,^9–14^ conjugated polymer-wrapping extraction^15–17^ and polymer aqueous two-phase extraction (ATP),^18–21^ utilize a difference in the adsorption and configuration of the dispersing agents depending on the structure and/or electronic properties of SWCNTs. DGU sorts SWCNTs by diameter, bandgap and electronic type *via* their density differences, which are closely related to the packing density due to the surfactant coverage, which consists of mixtures of bile salts and anionic-alkyl surfactants.^6,8^ Gel column chromatography elutes SWCNTs with a single chirality by the difference in the interaction between SWCNTs and the gel column medium. Sequential elution with different constitutions of mixed surfactant solution achieves a variety of chiralities.^9^ ATP with polyethylene glycol and dextran sorts single-chirality SWCNTs based on differences in hydrophobicity, which depends on the metallicity and affinity of the surface for specific surfactants.^18–20^ The charge on the surface of SWCNTs induced by pH and the addition of oxidant/reductant species is also important for the sorting of SWCNTs, and semiconducting (s-) SWCNTs and metallic (m-) SWCNTs have been successfully sorted by the optimization of charge on SWCNTs suspended with nonionic surfactants.^10,22^

Recently, we demonstrated a new approach for sorting s-SWCNTs by exploiting the phase transition of poly(*N*-isopropylacrylamide) (PNIPAM),^23^ a well-known thermoresponsive polymer, which shows reversible phase transitions in aqueous solutions at a lower critical solution temperature (LCST). Below LCST, PNIPAM is soluble in water because of intermolecular hydrogen bonding with water molecules at the carboxyl (C=O) and amine (N–H) groups in the polymer chain, whereas it forms globules above LCST by changing the hydrogen bonding manner to an intramolecular interaction between C=O and N–H.^24,25^ The intramolecular hydrogen bonding is known to form hydrophobic spacing inside the globule, which is separated from the aqueous solution by the polymer network; thus, the sequential aggregation of the globules makes a solid phase in the PNIPAM solution.^26,27^

We applied a solid/liquid phase separation to sort SWCNTs in the presence of NaClO, an oxidizing reagent, and succeeded in transferring s-SWCNTs to the liquid phase above the LCST of PNIPAM, while m-SWCNTs and some s-SWCNTs remained in the solid phase. The continuous use of PNIPAM *via* its reversible phase transition and the ultracentrifugation-free sorting procedure have a high potential for an economical sorting process that can be used for the mass production of SWCNTs with a specific electronic structure. However, the available surfactant is limited to bile acid, *e.g.*, sodium cholate (SC), because of the increase in the LCST of PNIPAM in the presence of other types of surfactants; therefore, knowledge of efficient single-chirality sorting with a mixed surfactant system^6,8,9,18^ is difficult to use for our sorting method.

In this study, we explored experimental conditions dedicated to the single-chirality sorting of SWCNTs *via* the phase transition in a thermoresponsive polymer. The interaction between PNIPAM and SC-coated SWCNTs is thought to be controllable by the difference in adsorption and configuration of SC to SWCNTs as well as the charge on the SWCNT surface. SWCNTs released into the liquid phase, *i.e.*, not captured in the solid phase, demonstrate selectivity in their structure and electronic properties by changing the above factors. Here, we demonstrated chirality-selective SWCNT sorting by controlling the concentration of PNIPAM, SC and additives.

## Results

### SWCNT sorting in the presence of sodium hypochlorite (NaClO)

**Fig. 1:**
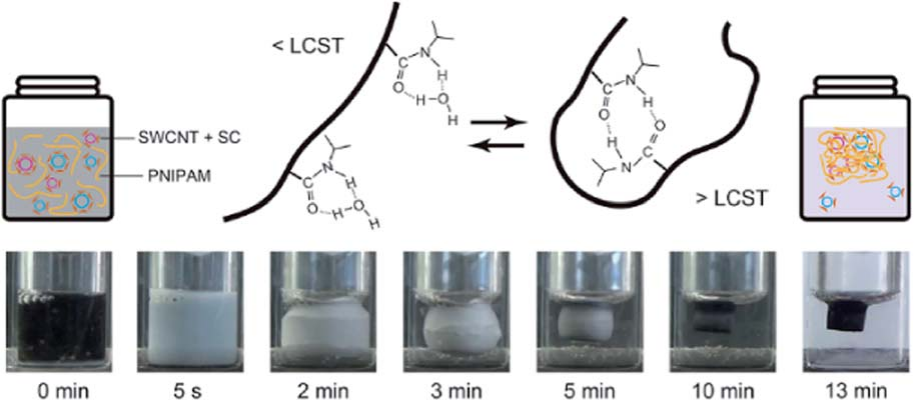
Schematic images and a diagram of the intermolecular and intramolecular hydrogen bonding interactions in a PNIPAM chain below the LCST (left) and above the LCST (right), respectively. Bottom row: images displaying the evolution of the aggregation and shrinkage of PNIPAM, including the dispersion of SWCNTs with SC.

A solution of SWCNTs dispersed in 2 wt% SC was added to 5 wt% PNIPAM aqueous solution, which was heated and maintained at a temperature above the LCST. 40 000 molecular-weight PNIPAM was used for this work, whereas 10 000 molecular-weight PNIPAM was used in the previous work.^23^ For the oxidation of m-SWCNTs, NaClO was added to the solution before heating. As shown in the photographs of Fig. 1, PNIPAM in the solution started to form globules just after heating, which formed a turbid solution and then became aggregates.^25,28^ Finally, the aggregate volume began to shrink, and the color of the gel changed from white to black.

**Fig. 2:**
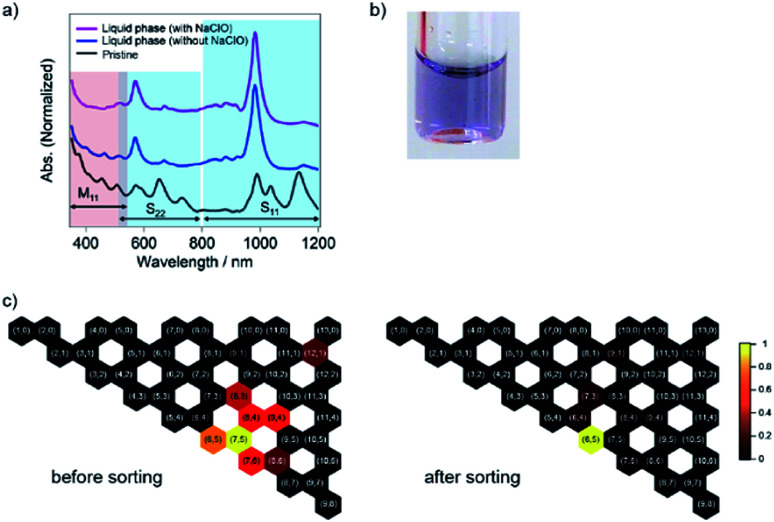
(a) Optical absorption spectra for the sorting of SWCNTs synthesized by the CoMoCAT method. The purple and blue lines correspond to the liquid phase after sorting with and without NaClO, respectively. The black line indicates the optical absorption spectrum of the pristine SWCNT solution. The blue and red shaded regions highlight the absorptions corresponding to the semiconducting SWCNTs (the first (S_11_) and second interband transitions (S_22_)) and the metallic SWCNTs (first interband transition (M_11_)), respectively. (b) Photograph of the obtained solution after sorting. (c) Observed SWCNT structures and their relative abundance as colored hexagons with (*n*,*m*) labels on a graphene lattice before (left) and after sorting (right). The color means the relative abundance of each chirality.

The liquid phase after the shrinkage of PNIPAM by heating exhibited absorption peaks at 571 nm and 985 nm, corresponding to the S_22_ and S_11_ transitions of the (6,5) nanotubes, respectively (Fig. 2).^5^ Compared with that of our previous report,^23^ the calculated purity of the (6,5) nanotubes was increased to 69% as the molecular weight of PNIPAM increased from 10 000 to 40 000. In addition, SWCNTs with larger diameters than the (6,5) nanotubes, such as (7,5) and (7,6), whose absorption peaks appeared at 1037 nm and 1134 nm, respectively, were preferably captured in the solid phase with 40 000 molecular-weight PNIPAM. In the absence of NaClO, the absorption peak corresponding to the m-SWCNTs was weakened but still appeared at ∼460 nm, while that mostly disappeared in the sorted sample with NaClO (the calculated purity of the whole amount of semiconducting SWCNTs was 96%). The partition coefficient was defined as *K*_(6,5)_ = *C*_l, (6,5)_/*C*_s, (6,5)_, where *C*_l, (6,5)_ and *C*_s, (6,5)_ are the concentrations of (6,5) nanotubes in the liquid and solid phases after the phase transition, respectively, and the value was determined as 0.1 by using the absorbance at 985 nm. Note that *C*_s, (6,5)_ was calculated by subtracting the absorbance of (6,5) nanotubes in the liquid phase from that of the pristine sample. The sorting yield was determined as 1.4% (see ESI†).

**Fig. 3:**
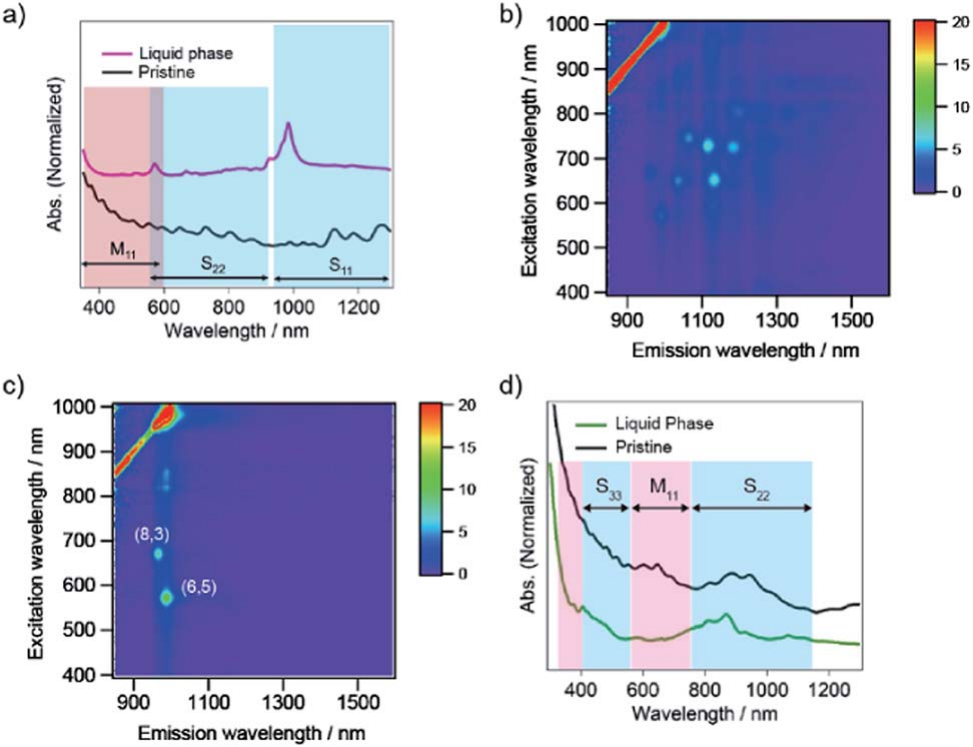
(a) Optical absorption spectrum corresponding to the liquid phase after the sorting of SWCNTs synthesized by the HiPco method. The black line indicates the optical absorption spectrum of the pristine SWCNT solution. PL excitation map for (b) the pristine solution and (c) the obtained liquid phase with HiPco tubes dispersed in D_2_O. The color indicates the intensity of the emission. The red stripe of high intensity is due to Rayleigh scattering of the excitation light. (d) Optical absorption spectrum corresponding to the liquid phase after the sorting of SWCNTs synthesized by the eDIPS method. The black line indicates the optical absorption spectrum of the pristine SWCNT solution. The blue and red shaded regions highlight the absorptions corresponding to the semiconducting SWCNTs and the metallic SWCNTs, respectively.

To explore the potential for the selective sorting of (6,5) nanotubes, SWCNTs with a diameter distribution of 0.8–1.2 nm and HiPco tubes, were sorted *via* the PNIPAM phase transition. As shown in Fig. 3a, the liquid phase exhibited absorption peaks at 571 nm and 985 nm, which were the same as those of CoMoCAT tubes. Note that the absorbance corresponding to the (6,5) tubes with HiPco tubes was lower than that with CoMoCAT tubes, as shown in Fig. 2, which is understood to be due to the difference in the amount of (6,5) tubes that constitute each sample. To confirm the single-chirality sorting of PNIPAM with HiPco tubes, excitation and emission contour maps of photoluminescence (PL) intensity for the pristine and sorting sample were demonstrated (Fig. 3b and c). A bright PL peak that showed an S_11_ emission at 987 nm and an S_22_ excitation at 570 nm appeared in the PL map, which corresponded to (6,5) nanotubes, although another PL peak corresponding to (8,3) was also observed. The similar diameter and S_11_ between (6,5) and (8,3) nanotubes is thought to cause the selective release of (6,5) nanotubes together with (8,3) nanotubes because of a small difference in the adsorption and configuration of SC on their sidewalls.

In the case of SWCNTs produced by the enhanced direct-injection pyrolytic synthesis (eDIPS) method with an average diameter of 1.3 nm,^29^ absorption at approximately 800–900 nm was observed in the liquid phase after the phase transition of PNIPAM (Fig. 3d), which corresponded to S_22_ of s-SWCNTs.^22^ The chirality candidates of the sorted s-SWCNTs were as follows: (9,7), (9,8), (10,5), (10,8), (10,9), (11,3), (11,6), (11,7), (12,1), (12,4), (12,5), and (13,2).^30,31^ The absorption in a range of 600–700 nm and 900–1100 nm, which corresponded to M_11_ and S_22_ of s-SWCNTs with a large diameter, respectively, was diminished after sorting, which indicated that small-diameter s-SWCNTs were selectively transferred to the liquid phase. The PNIPAM phase transition with SC-coated SWCNTs was understood to achieve diameter-selective sorting, and s-SWCNTs with small diameters in the sorting solution were released into the liquid phase in the presence of NaClO.

### PNIPAM concentration dependence

**Fig. 4:**
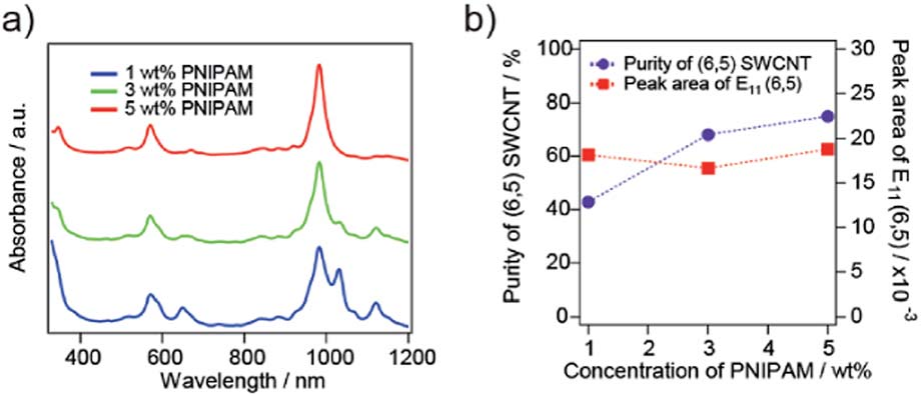
(a) Optical absorption spectra of the sorted liquid phase of the CoMoCAT SWCNTs with different concentrations of PNIPAM in solution. The PNIPAM concentrations are 1 wt% (blue), 3 wt% (green) and 5 wt% (red). (b) The calculated purity of (6,5) SWCNTs (purple circles with a dotted line) and the absorption peak area of the first interband transition of (6,5) SWCNTs (E_11_) (red squares with a dotted line).

The dependence on the PNIPAM concentration for the selective sorting of (6,5) nanotubes is shown in Fig. 4. As the concentration of PNIPAM increased, the purity of the (6,5) nanotubes increased and reached 69% with a 5 wt% PNIPAM solution. The partition coefficient of the (6,5) nanotubes was almost constant. The PNIPAM concentration of more than 10 wt% caused a reduction in the absorption peak intensity of (6,5) nanotubes (see ESI S2†). In the case of 1 wt% PNIPAM, SWCNTs with a large diameter, which exhibited S_11_ around a wavelength of 1000–1200 nm in Fig. 4a, were transferred to the liquid phase together with the (6,5) nanotubes, and their purity was reduced to 40%. A similar PNIPAM concentration dependence on the purity of (6,5) nanotubes was observed in the case of 10 000 molecular-weight PNIPAM as previously reported. Thus, the optimized PNIPAM concentration was 5 wt% for 40 000 molecular-weight PNIPAM, while the optimized PNIPAM concentration was 10 wt% for the molecular weight of 10 000.

### SC concentration dependence

**Fig. 5:**
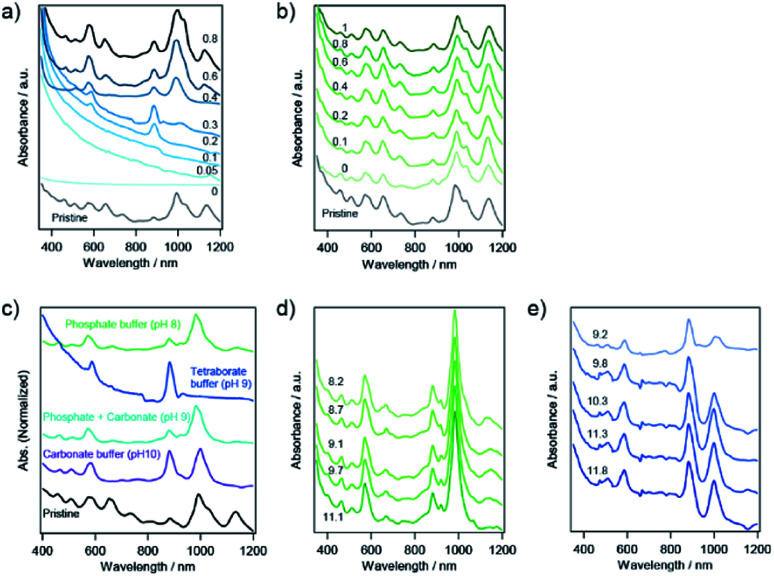
Optical absorption spectra of the pristine SWCNT suspension and the different fractions after sorting with NaClO concentrations in (a) 1 wt% SC and (b) 4 wt% SC, respectively. The value of NaClO indicates the dilution factor. (c) Optical absorption spectra of the pristine SWCNT suspension and the different fractions after sorting in the presence of different buffer solutions. The pH dependence of sorted SWCNTs with (d) phosphate buffer solution and (e) tetraborate buffer solution, respectively, demonstrated by changing pH with aqueous solution of sodium hydroxide. The value indicates pH of each solution.

The concentration of surfactant changes the micellar environment on an SWCNT, which is expected to change the adsorption and configuration of the dispersant molecules on the SWCNT as well as the affinity of circumambient molecules for the micellar SWCNTs. In the case of 1 wt% SC, whole dispersed SWCNTs were captured in the solid phase in the absence of NaClO because no absorption peaks related to SWCNTs were observed in the liquid phase (Fig. 5a). With an increase in the concentration of NaClO in 1 wt% SC, the liquid phase exhibited weak absorption peaks related to s-SWCNTs with a smaller diameter. In the case of 4 wt% SC, the same absorption spectrum as the pristine solution was obtained. The increase in the concentration of NaClO caused no change in the absorption peaks.

### pH dependence

**Fig. 6:**
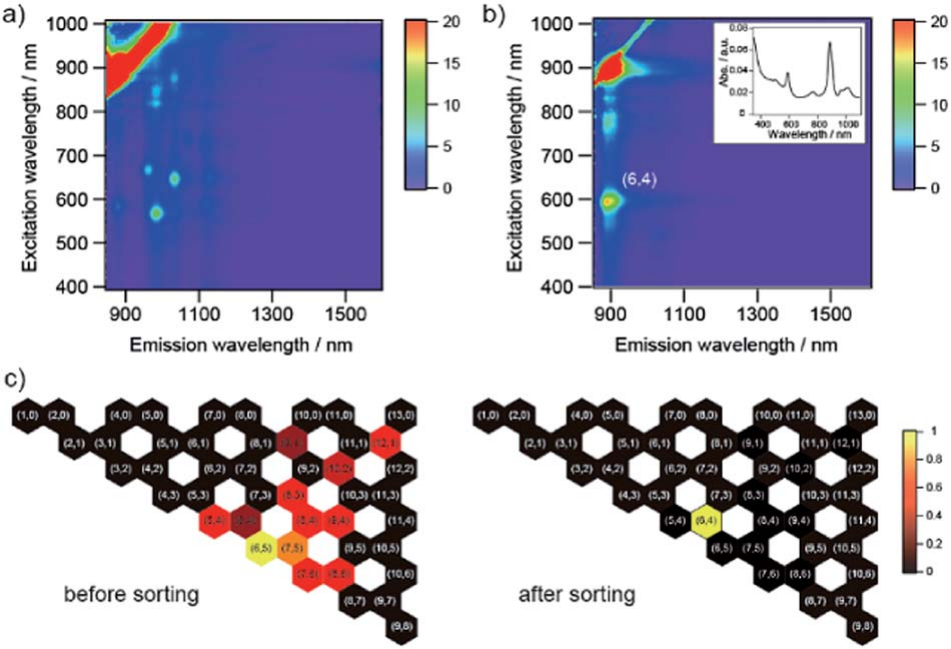
PL excitation map for (a) before and (b) after sorting of SWCNTs synthesized by the CoMoCAT method in the presence of a tetraborate buffer solution in D_2_O. The color indicates the intensity of the emission. The red stripe of high intensity is due to Rayleigh scattering of the excitation light. (Inset) Optical absorption spectra for the sorting of SWCNTs synthesized by the CoMoCAT method in the presence of a tetraborate buffer solution in D_2_O. There are no PL peaks in the region of the white panel. (c) Observed SWCNT structures and their relative abundance as colored hexagons with (*n*,*m*) labels on a graphene lattice before (left) and after sorting (right). The color means the relative abundance of each chirality.

The charge on the SWCNT surface is known to be changed by pH, and the hydration of PNIPAM is also affected by pH. To investigate the effect of pH on the sorting of SWCNTs with PNIPAM, we used three different buffer constituents: phosphate buffer solution (pH 8), tetraborate buffer solution (pH 9), and carbonate buffer solution (pH 10). The selective sorting of SWCNTs with small diameters was observed at pH 8, and m-SWCNTs existed in the liquid phase due to the absence of NaClO (Fig. 5c), and the same sorting result was obtained at pH 10. On the other hand, the sorting solution at pH 9 exhibited two absorption peaks at 589 nm and 889 nm alone, corresponding to the S_22_ and S_11_ transitions of (6,4) nanotubes, respectively. The addition of a pH 9 solution, which was prepared by mixing the phosphate buffer solution and carbonate buffer solution, had no positive effects on the sorting, and the obtained absorption spectrum exhibited the same feature as that at pH 8. The pH dependence on the sorting with a single buffer solution (Fig. 5d and e) also exhibited no positive effects of pH on the sorting. The calculated purity of (6,4) nanotubes sorted by the addition of tetraborate buffer solution was 98%. The partition coefficient was defined as *K*_(6,4)_ = *C*_l, (6,4)_/*C*_s, (6,4)_, and the value was determined as 0.37 by using the absorbance at 889 nm. A bright PL peak that showed an S_11_ emission at 883 nm and an S_22_ excitation at 590 nm appeared in the PL map, which corresponded to (6,4) nanotubes, as shown in Fig. 6. These results indicate that the pH in the prepared solution for sorting had no effect on the sorting of (6,4) nanotubes, but the constituent chemical in the tetraborate buffer solution was key to selectively releasing (6,4) nanotubes to the liquid phase after the phase transition of PNIPAM.

### SWCNT sorting in the presence of sodium salt

**Fig. 7:**
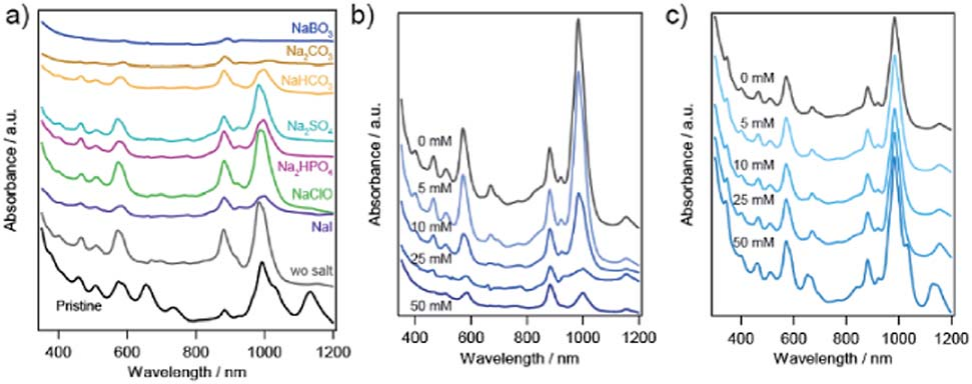
(a) Optical absorption spectra of the pristine SWCNT suspension and the different fractions after sorting in the presence of different sodium salt aqueous solutions as well as in the absence of sodium salt. The salt concentration dependence of sorted SWCNTs with (b) NaBO_3_ and (c) Na_2_SO_4_, respectively.

The effect of sodium salt on the sorting of SWCNTs with PNIPAM was investigated, as shown in Fig. 7. The absorption spectra in addition to Na_2_SO_4_ and NaClO were similar to those in the absence of sodium salt. Note that the lower concentration of a diluted NaClO aqueous solution with the dilution factor of 0.008 were used for the experiment to approximate the NaClO concentration to 10 mM. In addition to NaI or NaHCO_3_, two major absorption peaks still appeared in the sorted solution, although the ratio of the absorption peak of (6,4) nanotubes to that of (6,5) nanotubes was increased compared to the sorting result in the absence of sodium salt. When Na_2_CO_3_ or NaBO_3_·4H_2_O was added to the prepared solution, the (6,4) nanotubes were selectively released to the liquid phase, and the obtained solution exhibited absorption peaks related to the S_11_ and S_22_ of (6,4) nanotubes. M-SWCNTs were present in the all samples presented in Fig. 7.

The prepared solution in the presence of Na_2_CO_3_ should be heated as soon as adding the salt, or else (6,5) nanotubes would be sorted together with (6,4) nanotubes after incubating the solution for a few minutes. The controlled experiments without PNIPAM indicate that the absorption peaks correlating to S_11_ of large diameter s-SWCNTs were red-shifted and broadened in the case of Na_2_CO_3_ (see ESI S3†). The incubating time dependence in Na_2_CO_3_ would be caused by the change in the configuration of SC on the sidewall of SWCNTs in the presence of Na_2_CO_3_.

## Discussion

### Sorting mechanism

The interaction between the PNIPAM and SC–SWCNT complex is understood to orchestrate the sorting of s-SWCNTs with a small diameter by using the thermoresponsive phase transition. SC is proposed to cause a change in the interaction with SWCNTs depending on their electronic properties and diameter: (i) m-SWCNTs with high polarizability strongly interact with SC compared with that of s-SWCNTs, and (ii) SC favorably desorbs from the sidewall of SWCNTs with a large diameter due to their low activation energy.^32^ Research on the DGU sorting of SWCNTs with SC and sodium dodecyl sulfate (SDS) reveals that the adsorption of SDS on a vacant area of the sidewalls with nonuniform SC coverage and the desorption of SC with the subsequent adsorption of SDS produces different buoyant densities.^6,8^ PNIPAM is expected to weakly interact with SWCNTs because of the unsuccessful dispersion of SWCNTs with a PNIPAM aqueous solution. Thus, the desorption of SC from the sidewall of SWCNTs and the subsequent absorption of PNIPAM is an unlikely event in our sorting method.

**Fig. 8:**
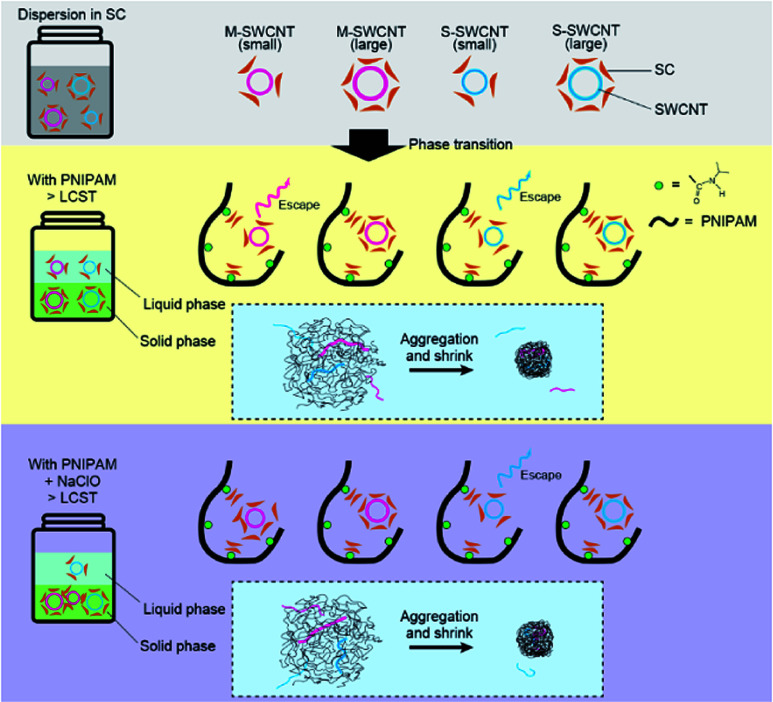
Schematic interpretation of the sorting mechanism for an s-SWCNT with a small diameter by PNIPAM.

The presence of SC in a PNIPAM solution is reported to lower the LCST of PNIPAM due to the influence on the aggregation of the polymer.^33^ SC is known to form a dimer in an aqueous medium even at concentrations below its critical micellar concentration (10–15 mM); therefore, the hydrophobic faces of SCs, a convex side (β-plane), come together.^34^ The SC dimer is expected to interact with the hydrophilic side of PNIPAM in the sorting solution before heating, which attracts the micellarized SC on the sidewall of SWCNTs. This hypothesis explains the selective sorting of s-SWCNTs with a small diameter *via* the PNIPAM phase transition (Fig. 8).

Small-diameter SWCNTs are expected to adsorb fewer SC molecules on their sidewall due to their large curvature, which causes less interaction with SC-decorated PNIPAM and results in the failure of capturing SWCNTs in the aggregation of PNIPAM. In the presence of NaClO, m-SWCNTs and s-SWCNTs with a small energy bandgap are hole-doped,^18^ which increases the rate of subsequent SC adsorption around the micellized SWCNTs due to an increase in their polarizability. Then, m-SWCNTs can be captured in the aggregation of PNIPAM, and s-SWCNTs with only a small diameter are understood to be released in the liquid phase after the phase transition of PNIPAM.

The SC concentration dependence on sorting by PNIPAM is also explained as follows. In a low concentration of SC, the probability of PNIPAM interacting with SC-micellized SWCNTs is thought to increase due to less SCs in the space that the PNIPAM and SWCNTs surround; thus the above conditions result in an increase in the probability of capturing the micellized SWCNTs inside the PNIPAM globule. At high concentrations, the increased amount of SCs in the space impedes the interaction of PNIPAM with SC-micellized SWCNTs, resulting in a decrease in the selectivity of s-SWCNTs with a small diameter.

The pH variation in a base region is thought to cause a small change in the difference of zeta potential on the m- and s-SWCNTs. Therefore, pH produces no positive effects on sorting due to a slight change in the arrangement of SC on the sidewall of SWCNTs, which depends on their electronic structures.

### The effect of sodium salt on the sorting of SWCNTs

The LCST of PNIPAM in an aqueous solution is known to change depending on the existing anionic and cationic species.^35^ The systematic order of ions follows the Hofmeister series, which is known as the ability to precipitate proteins and was formulated in 1888.^36^ A typical series of anions is as follows: CO_3_^2−^ > SO_4_^2−^ > S_2_O_3_^2−^ > H_2_PO_4_^−^ > F^−^ > Cl^−^ > Br^−^ ≈ NO_3_^−^ > I^−^ > ClO_4_^−^ > SCN^−^ when arranged in decreasing order of kosmotropicity.^35,37–39^ The LCST of PNIPAM in an ionic liquid has been reported to be reduced in the presence of a kosmotropic anion, which indicates that kosmotropic anions strongly bind to water molecules. The strong bond stabilizes the aggregation of PNIPAM and forms a compact structure due to the enhancement in the hydrophobic collapse of PNIPAM.^40^ Thus, the dependence of anions on the sorting of SWCNTs is explained by kosmotropicity. Kosmotropic anions, such as CO_3_^2−^, promote the dehydration of PNIPAM, which causes a stronger interaction between PNIPAM and the SC-micellized SWCNTs than those of chaotropic anions, such as I^−^. The presence of the sodium sulfate and phosphate buffer solutions have no effect on the selective sorting of SWCNTs, indicating that a very strong kosmotropic anion, with a small ionic radius and high charge density,^39^ seems to be effective for the sorting of SWCNTs by PNIPAM.

## Experimental

### Method

The basic sorting procedure for s-SWCNTs *via* the phase transition of PNIPAM has been described in a previous report. A 9 mg portion of SWCNTs was dispersed in 10 g of distilled water containing 2.0 wt% SC (FUJIFILM Wako Pure Chemical Corp., Japan) using a probe tip ultrasonicator (TOMY, UR-20R, Japan) for over 1.5 h in a cold bath. The dispersion was then centrifuged (TOMY, Suprema 21, Japan) at 16 210*g* for 3 h with a swing rotor. A 50 μL aliquot of the SWCNT dispersion and 10 μL of a diluted NaClO (active chlorine of 8.5–13.5%; Nacalai Tesque Inc., Japan) aqueous solution with the dilution factor of 0.06 were mixed in an Eppendorf tube with a vortex mixer, which was followed by mixing with 200 μL of a 5 wt% PNIPAM (avg. MW: 40 000, Polysciences Inc., USA) aqueous solution. The prepared solution was heated to 45 °C and incubated for 15 min. After the phase transition, the liquid phase was collected. CoMoCAT (Signis CG100, Sigma-Aldrich, USA; diameter: 0.7–1.3 nm) and HiPco tubes (Raw, Nanointegris, USA; diameter: 0.8–1.2 nm) were used as received. SWCNTs produced by the eDIPS method were also used, and their average diameter was characterized as 1.3 nm by Raman and optical absorption spectroscopy.

For the investigation of pH and sodium salt dependences on sorting, purchased aqueous buffer solutions or prepared 10 mM sodium salt solutions were used instead of the NaClO aqueous solution and consisted of the following: 0.1 M phosphate buffer solution (pH 8.0; FUJIFILM Wako Pure Chemical Corp., Japan), 0.38% tetraborate buffer solution (pH 9.18, Horiba, Ltd., Japan), 0.025 mol kg^−1^ carbonate buffer solution (pH 10.01; Nacalai Tesque Inc., Japan), sodium sulfate, sodium iodide, sodium carbonate, and sodium hydrogen carbonate (FUJIFILM Wako Pure Chemical Corp., Japan).

### Characterization

Optical absorption spectra of the obtained samples were collected on a UV-Vis-NIR spectrophotometer (UV-3600, SHIMADZU corp., Japan) with a 10 mm path length semi-microcuvette. The PL excitation and emission contour maps were characterized by using a Shimadzu NIR-PL system (CNT-RF) equipped with a liquid N_2_-cooled InGaAs detector array. A 10 nm slit width was used for both excitation and emission. Excitation light was generated by a xenon arc lamp with an exposure time of 5 s. The pH of each sample was measured by a pH meter (LAQUAtwin-pH-33B, Horiba, Ltd., Japan).

## Conclusions

The sorting technique for chirality-selective SWCNTs exploits the hydrophobic collapse of PNIPAM with the formation of intramolecular hydrogen bonding at the C=O and N–H groups and the subsequent capture of SC-micellized SWCNTs. The interaction of SC-decorated PNIPAM with SC-micellized SWCNTs and the surface coverage of SC on the sidewall of SWCNTs differ according to the size and metallicity of SWCNTs, which causes the selective release of SWCNTs into the liquid phase after the PNIPAM phase transition. Anions in a sorting solution also affect the sorting of SWCNTs, and we achieve the selective sorting of (6,4) nanotubes by adding strong kosmotropic anions, such as tetraborate and carbonate. This sorting technique has some advantages to scale up the production of SWCNTs: a simple sorting scheme with a centrifugation-free process, less contamination due to the liquid–solid phase separation of PNIPAM and continuous sorting by the recycling use of PNIPAM. The progress presented in this work will enable the mass production of SWCNTs with a single chirality, which should accelerate the use of SWCNTs, with homogeneous electronic properties, in electronic devices and sensor applications.

## Conflicts of interest

There are no conflicts to declare.

## Supplementary Material

RA-010-D0RA04357E-s001

RA-010-D0RA04357E-s002
