# Supplementary material for: Role of constituents for the chirality isolation of single-walled carbon nanotubes by the reversible phase transition of a thermoresponsive polymer
Source: RSC Adv. 2020 Jun 29;10(41):24570–6. doi: 10.1039/d0ra04357e (PMC9055163; doi:10.1039/d0ra04357e)
Supplement: RA-010-D0RA04357E-s001 [file RA-010-D0RA04357E-s001.pdf]

Electronic Supplementary Material (ESI) for RSC Advances.
This journal is © The Royal Society of Chemistry 2020

Supplementary Information for

Role of Constituents for the Chirality Isolation of Single-Walled Carbon Nanotubes by the Reversible Phase Transition of a Thermoresponsive Polymer

Eriko Shimura,[a] *Tomomi Tanaka,*[a] *Yuki Kuwahara,*[b] *Takeshi Saito,*[b] *Toshiki Sugai* [a] *and Shota Kuwahara* [a]*

[a] Department of Chemistry, Faculty of Science, Toho University, 2-2-1 Miyama, Funabashi, Chiba, 274-8510, Japan

[b] Nanomaterials Research Institute, National Institute of Advanced Industrial Science and Tchnology, Tsukuba 305-8565, Japan

*syouta.kuwahara@sci.toho-u.ac.jp

Quantitative analysis of the purity of sorted (6,5) SWCNTs.

The purity of (6,5) nanotubes in the liquid phase was quantitatively calculated by comparing the S_{11} absorption peak area of the (6,5) nanotubes with the sum of the absorbance in a range of 800–1200 nm. The optical absorption by the SWCNTs in the UV region, which can be fitted to a Lorenz function, overlaps with the absorption peaks in the visible-NIR region that correspond to the interband electronic transitions in the SWCNTs; therefore, they must be subtracted from the obtained absorption spectra to assess the purity of the (6,5) nanotubes. The Lorenz function for subtracting the overlaid background absorption in the visible to NIR region has a peak at 4.6 eV (λ = 270 nm), and the parameters in the function are set so that no absorbances below a zero value are obtained in the whole region after subtracting the background absorption.

The absorption peak area of S_{11} for the (6,5) nanotubes and the total absorbance in a range of 800–1200 nm were calculated by multiplying the absorbance by the energy range of photons as follows:

$$I = \sum_{i} A(E)\Delta E \qquad (1)$$

where I is the total area of optical absorption, $A(E)$ is the absorbance at the photon energy (eV), and ΔE is the photon energy (eV). The purity of the (6,5) nanotubes was calculated by using the following equation:

$$(\text{purity}) = \frac{I_{(6,5)}}{I(800-1200\,\text{nm})} \times 100(\%) \qquad (2)$$

Quantitative analysis for the relative abundance of the sorted sample (Fig. S2).

First, the optical absorption by SWCNTs in the UV region was subtracted as described above. Then, the absorption region corresponding to the first interband transition (S_{11}) was fitted by a sum of Lorenz functions:

$$f(E) = y_0 + \sum \frac{a_{(n,m)}}{(E - S_{11}(n,m))^2 + b_{(n,m)}} \tag{3}$$

The absorption peak area of S_{11} for each chirality was calculated by multiplying the absorbance by the energy range of the photons as follows:

$$I(n,m) = \sum_i \left(\frac{a_{(n,m)}}{(E - S_{11}(n,m))^2 + b_{(n,m)}} \right) \Delta E \tag{4}$$

The relative abundance of each chirality was calculated by using the following equation:

$$(\text{relative abundance}) = \frac{I(n,m)}{\sum I(\mathrm{n,m})} \tag{5}$$

Partition coefficient of the sorted (6,5) SWCNTs.

The absorption peak area of S_{11} for the (6,5) nanotubes was calculated by using equation 1. The partition coefficient $K_{(6,5)}$ of the sorted (6,5) SWCNTs was obtained by using the following equation:

$$K_{(6,5)} = \frac{I_{l,(6,5)}}{\frac{I_{pr,(6,5)}}{D_{pr}} - I_{l,(6,5)}} \tag{6}$$

where *l* is the liquid phase of the sorted sample, *pr* is the pristine sample, *I* is the total area of optical absorption, and *D* is the dilution rate.

Sorting yield of the sorted (6,5) SWCNTs.

The sorting yield (η) was calculated as following,

$$\eta(\%) = \frac{C_{l,(6,5)}}{C_{pr}} \times 100 \tag{7}$$

$$C_{pr} = \left(C_{l,(6,5)} + C_{s,(6,5)}\right) \times \frac{1}{P_{pr,(6,5)}} \times \frac{1}{P_{pr,semi}} \tag{8}$$

where C_{pr} is the concentrations of (6,5) nanotubes in the pristine sample for the sorting, $P_{pr,\,(6,5)}$ and $P_{pr,\,semi}$ are the purity of (6,5) nanotubes and s-SWCNTs in the pristine, respectively. The calculated $P_{pr,\,(6,5)}$ was 0.18 by comparing the absorption peak area in a range of 800–1200 nm, and the value of 0.80 was assumed for $P_{pr,\,semi}$ as previously reported.[41] Then, the sorting yield was determined as 1.4%.

Supplementary Reference:

41. S. Kuwahara, Y. Kuwahara and H. Shinohara, *J. Nanomater.*, 2014, **2014**, 262940.

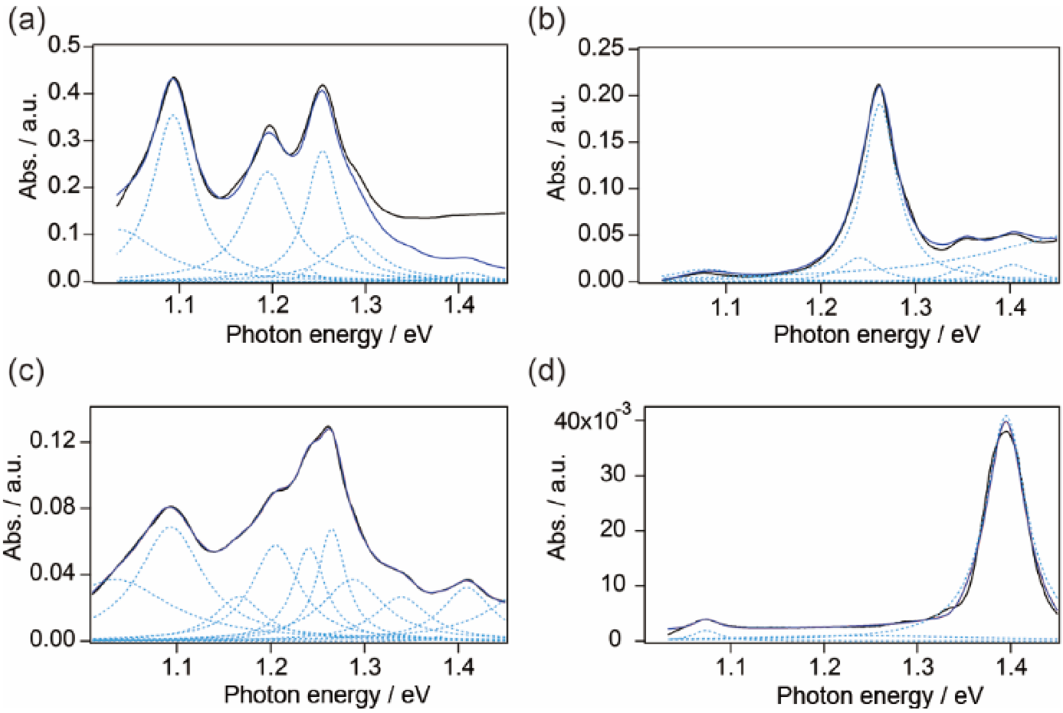

Fig. S1 Optical absorption spectra (black solid line) and a sum of Lorentzian peaks (blue dotted lines) for (a) the pristine SWCNT solution and (b) the sample sorted in the liquid phase that was used for calculating the relative abundance shown in Figure 2c. (c) and (d) are optical absorption spectra (black solid line) and a sum of Lorentzian peaks (blue dotted lines) for the pristine SWCNT solution and the sample sorted in the liquid phase that was used for calculating the relative abundance shown in Figure 6c, respectively.

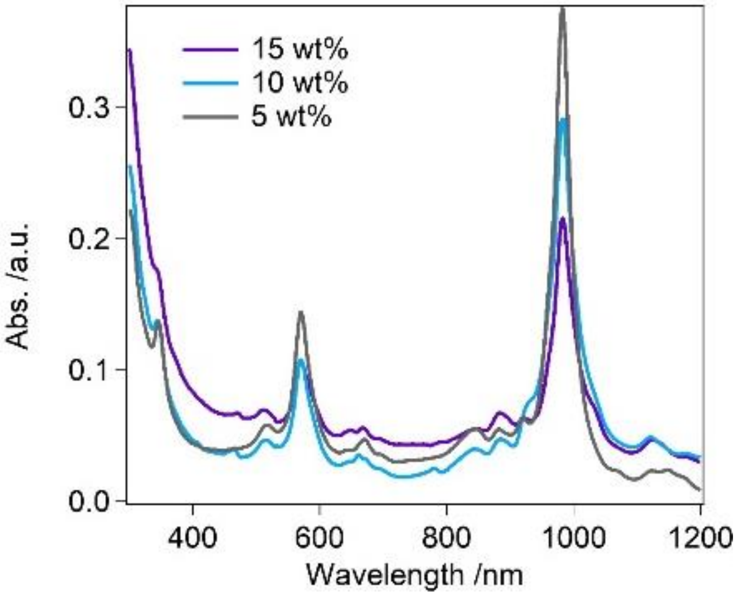

Fig. S2 Optical absorption spectra of the sorted liquid phase of the CoMoCAT SWCNTs with different concentrations of PNIPAM in solution. The PNIPAM concentrations are 5 wt% (gray), 10 wt% (blue) and 15 wt% (purple).

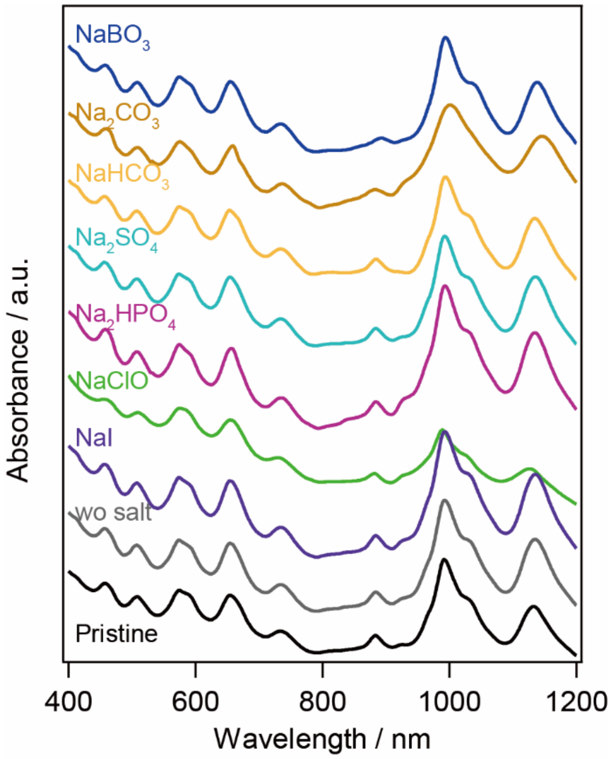

Fig. S3 Optical absorption spectra of SWCNT dispersions in the presence of different sodium salt aqueous solutions as well as in the absence of sodium salt. A 50-μL aliquot of the SWCNT dispersion in 2 wt% SC, 10 μL of a 10 mM sodium salt aqueous solution and a 200 μL of distilled water were mixed. The prepared solution was heated to 45 °C and incubated for 15 min.
